# Pedobarography as a clinical tool in the management of diabetic feet in New Zealand: a feasibility study

**DOI:** 10.1186/s13047-017-0205-6

**Published:** 2017-06-09

**Authors:** Jason K. Gurney, Uwe G. Kersting, Dieter Rosenbaum, Ajith Dissanayake, Steve York, Roger Grech, Anthony Ng, Bobbie Milne, James Stanley, Diana Sarfati

**Affiliations:** 1 Cancer and Chronic Conditions (C3) Research Group, Department of Public Health, University of Otago, Wellington, New Zealand; 2 Center for Sensory-Motor Interaction, Aalborg University, Aalborg, Denmark; 3 University Hospital Muenster, Muenster, Germany; 4 Counties Manukau District Health Board, Auckland, New Zealand; 5 Northland District Health Board, Whangarei, New Zealand

**Keywords:** Diabetes, Pedobarography, Lower-limb complications, Ulceration, Plantar pressure

## Abstract

**Background:**

The peripheral complications of diabetes mellitus remain a significant risk to lower-limb morbidity. In New Zealand, risk of diabetes, comorbidity and lower-limb amputation are highly-differential between demographic groups, particularly ethnicity. There is growing and convincing evidence that the use of pedobarography – or plantar pressure measurement – can usefully inform diabetic foot care, particularly with respect to the prevention of re-ulceration among high-risk patients.

**Methods:**

For the current feasibility study, we embedded pedobarographic measurements into three unique diabetic foot clinic settings in the New Zealand context, and collected pedobarographic data from *n* = 38 patients with diabetes using a platform-based (Novel Emed) and/or in-shoe-based system (Novel Pedar). Our aim was to assess the feasibility of incorporating pedobarographic testing into the clinical care of diabetic feet in New Zealand.

**Results and Conclusions:**

We observed a high response rate and positive self-reported experience from participants. As part of our engagement with participants, we observed a high degree of lower-limb morbidity, including current ulceration and chronic foot deformities. The median time for pedobarographic testing (including study introduction and consenting) was 25 min. Despite working with a high-risk population, there were no adverse events in this study. In terms of application of pedobarography as a clinical tool in the New Zealand context, the current feasibility study leads us to believe that there are two avenues that deserve further investigation: a) the use of pedobarography to inform the design and effectiveness of offloading devices among high-risk diabetic patients; and b) the use of pedobarography as a means to increase offloading footwear and/or orthoses compliance among high-risk diabetic patients. Both of these objectives deserve further examination in New Zealand via clinical trial.

**Electronic supplementary material:**

The online version of this article (doi:10.1186/s13047-017-0205-6) contains supplementary material, which is available to authorized users.

## Background

Diabetes mellitus is a metabolic dysfunction characterised by high concentrations of glucose within blood (termed *hyperglycaemia*), which is caused by deficits in insulin production and activity [1, 2] and/or cellular resistance to insulin [3]. One of the most common complications of diabetes is *peripheral neuropathy* [4], which involves damage to and dysfunction of peripheral nerves – starting at the extremities of the limbs, then progressing towards the torso [5]. A loss of peripheral sensory function – and thus pain signalling [6] – compounded by autonomic and neuromuscular complications [7, 8] increases the risk of foot ulceration due to trauma or repetitive loading of the plantar surface of the foot [9]. If left untreated, these ulcers can become infected – and due to reduced healing capacity [10], infected wounds can become gangrenous and lower-limb amputation may ultimately be required [7].

Patients with diabetes are 15 times more likely to require lower-limb amputation than people without diabetes [5, 11], and 15% of those with diabetes and peripheral neuropathy will require foot amputation [12, 13]. The vast majority (80%) of lower-limb amputations among patients with diabetes are preceded by a foot ulcer [9]; thus, primary intervention to prevent foot ulceration among these patients – and secondary intervention that assists in expedient healing of current ulcers – is a highly-desirable goal for both the patient and health care services.

Pressures beneath the plantar surface of the foot are increased in the diabetic foot compared to healthy populations [14–17]. These increases in pressure are the result of a combination of morphological, muscular and sensory abnormalities [7, 14, 18–21]. For example, ‘clawing’ of the toes is common among the diabetic population [7], as is the deformity known as *hallux valgus* [22, 23] which arises in one-third of all patients with diabetes [24] due to weakening of intrinsic foot musculature in the hallux region [25]. These foot and toe deformities ultimately lead to localised increases in plantar pressure, particularly at the metatarsal heads [14]. Crucially, there is a high correlation between elevated plantar pressure and foot ulceration [15, 26–29].

Plantar pressure and the dynamic structure of the foot can be measured using a technique called *pedobarography* – in which measurements of plantar pressure are taken during walking, either via insole-based [30] or platform-based systems [31]. Pedobarographic measurements provide a clinically-relevant quantification of the stress that small areas of the plantar surface are experiencing during barefoot or shod walking, and also enable identification of any abnormalities in dynamic foot structure that may be causing these elevated plantar pressures [21].

Evidence to date suggests that the use of pedobarography can have a profound impact on the prevention of ulceration among patients with diabetes. In 2013, Bus et al. [32] observed that among patients with a recently-healed plantar ulcer who had high adherence to their treatment, the use of in-shoe pressure measurements to guide footwear customisation substantially reduced the risk of ulcer reoccurrence compared to those who received footwear that was not modified based on this data (re-ulceration odds ratio: 0.38, 95% CI 0.15–0.99) [32]. In 2014, Ulbrecht et al. [33] conducted a randomised controlled trial (RCT) within a diabetic foot clinic setting, randomly assigning patients with recently-healed ulcers into two treatment groups: those for whom plantar pressure measurement was used to inform the design and construction of orthoses that offload the site of the ulcer (intervention group), and those who received standard orthoses (control group). The premise of the intervention was that orthoses designed with plantar pressure information would result in better offloading of high-pressure areas of the foot [34]. Astonishingly, the authors observed that the rate of re-ulceration among patients in the control group was three-and-a-half-times higher than the intervention group (re-ulceration proportions: intervention group 9.1%, control group 25.0%; adjusted hazard ratio [HR]: 3.4, 95% CI 1.3–8.7). This evidence suggests that there is an opportunity for pedobarography to customise treatment plans and substantially improve outcomes among high-risk patients with diabetes [33].

Pedobarography as a clinical tool is not just limited to assisting construction of offloading orthoses: it is possible that screening patients with diabetes for the presence of high plantar pressures can prevent ulceration by prompting clinical attention toward areas of high pressure that otherwise may have gone unnoticed (including initiating efficacious treatment, such as debridement). There is some evidence that pedobarography has a relatively high degree of specificity (and a moderate degree of sensitivity) in identifying those at risk of developing a foot ulcer [35].

There are several other potential pathways by which pedobarography may reduce risk of diabetic foot ulceration: for example, it is possible that pedobarography may provide an effective source of biofeedback for patients – whereby patients are provided with information regarding peak pressures beneath their feet, and then alter potentially-harmful behaviour as a result (e.g. wearing adequate footwear) [36]. Our understanding of the potential impact of pedobarography as a biofeedback tool is still in its infancy.

### Diabetes in the New Zealand context

Diabetes is a common chronic condition in New Zealand – with more than 6% (or >220,000 people) of the adult population diagnosed with the disease [37]. The prevalence of diabetes is not evenly distributed across the population [38, 39]: while the majority European population has a diabetes prevalence of 5%, it is estimated that more than 7% of indigenous Māori and 13% of the Pacific Island population are affected by this disease [37]. Māori not only carry an inequitable burden of diabetes, but are also more likely to experience other serious comorbidity (such as cardiovascular [40] and renal disease [41]) – and have rates of lower-limb amputation that are 84% higher than those experienced by non-Māori/non-Pacific/non-Asian patients with diabetes (adjusted HR: 1.84, 95% CI 1.54–2.19) [42]. Pacific New Zealanders with diabetes appear no more likely to require lower-limb amputation than New Zealand Europeans [42]. The reasons for this disparity in amputation risk remain obscure.

In summary, there is a burgeoning body of evidence that suggests pedobarography can play a role in preventing serious limb- (if not life-) threatening complications among patients with diabetes. However, the efficacy of such an intervention remains scantily explored in clinical trials, and is entirely untested in the New Zealand context. The aim of the feasibility study described here was to begin to address this information gap by answering the following questions:

- What is the response rate among patients invited to take part in pedobarographic testing?
- To what extent does the additional testing interrupt normal clinic time?
- What is the experience of patients who take part in pedobarographic testing?
- What applications of pedobarographic testing are most useful to those clinicians in charge of diabetic foot care?
- What aspects of pedobarography could feasibly be tested in a clinical trial?
- What unique issues exist in NZ that impact the usefulness of pedobarography as a tool in the care of the diabetic foot, and/or will need to be taken into consideration during a full clinical trial? For example, what type of footwear (closed/non-closed) do patients routinely wear to clinical appointments?

The current manuscript describes our observations with respect to these questions.

## Methods

### Data collection setting

Pedobarographic testing was offered to patients attending outpatient diabetic foot clinics in three separate locations in the northern part of New Zealand. The clinics were: Manukau Superclinic (South Auckland), Whangarei Hospital High-Risk Foot Clinic (Whangarei), and the Bay of Islands Hospital High-Risk Foot Clinic (Kawakawa). The South Auckland and Whangarei clinics are located in metropolitan areas, while the Kawakawa clinic is located in a rural area. Data collection occurred over eight separate clinic days between 9th November to 25th November 2016.

The diabetic foot clinics were a mixture of a) high-risk clinics treating patients with current or healing ulcers (three clinic days), b) moderate-risk clinics treating patients requiring foot assessment but without a current or healing ulcer (three clinic days), and c) general diabetes clinics at which patients receive a general assessment of their diabetic health (two clinic days).

### Participants

Potential participants were patients with Type-1 or Type-2 diabetes who were attending clinics as part of their normal diabetes care, referred by their diabetes clinicians. Only patients who were able to walk without pain were invited in to the study (e.g. those in wheelchairs were not included). The total number of patients who were invited in to the study, as well as the number of patients who declined (and the reason given for declining), was derived from self-report from the referring clinicians.

A total of 48 patients were invited to participate in the study by referring clinicians, of whom 39 agreed (response rate: 81% of those invited). The most common reason for declining to participate in the study was time pressure (*n* = 7; 77% of those who declined). One patient began pedobarographic testing but stopped due to walking difficulty, and thus was excluded from further analysis. Therefore, the final study group included 38 participants.

Those who agreed to participate in the study were taken to meet the pedobarography team (JG, UK, DR), at which point the clinician provided the study team with the clinical characteristics of the patient, including the presence of peripheral neuropathy, foot deformity, previous or current ulceration, amputation(s) and other relevant lower-limb complications. The clinician also stated what information they hoped to gain from the pedobarographic measurement. The patient was taken by a team member (JG) to a quiet area to discuss what was involved in participation, and to gain written informed consent. Once the consent process was completed, pedobarographic measurements began.

### Demographic data collection

Participant age in years was provided by the referring clinician, as derived from clinical records. Participant ethnicity was self-identified using the ethnicity categorisations used in the 2013 New Zealand Census, in which a participant can choose multiple ethnic affiliations. Ethnicity data were aggregated into New Zealand European, Māori, Pacific Island and non-New Zealand European/Māori/Pacific for reporting.

### Pedobarography measurements

Pedobarographic measurements were performed with either a platform-based system (Emed AT, 2 sensors/cm^2^, Novel GmbH, Munich, Germany) and/or an insole-based system (Pedar X, Novel, Munich Germany). A decision whether to use the platform-based system, in-shoe based system or both was made in consultation with the clinician. This decision was made based on what information the clinician hoped to gain from the pedobarographic measurement; for example, if the clinician was hoping to ascertain whether a recently-introduced offloading device was actually reducing loads around a site of a current ulceration, the in-shoe system was used. In many cases, the clinician was interested in comparing conditions (hereafter termed ‘multiple conditions’); for example, in-shoe pressures using a standard orthotic insert compared to a customised orthotic insert, or barefoot pressures before and after plantar debridement. If only one condition was of interest (e.g. only barefoot pressure), this was termed ‘single condition’. The system-specific method of data collection is described below for each system type.

Data were collected within the Novel Emed and/or Novel Pedar data collection software. For the platform-based system, participants were instructed to walk barefoot over an 8 m foam runway, with the Emed platform located in the middle of the runway. Participants were instructed to walk at normal pace and to not aim for the platform as they walked. A minimum of three steps were taken before and after contacting the platform [43]. Five trials were collected per foot (one trial = one foot) for each participant [44–46], although for some participants only three trials per foot could be practically collected, primarily due to participant fatigue and difficulty in striking the platform.

For the insole-based system, participants were fitted with the correct insoles for their shoe size (most commonly size EU 42 insole). Footwear was not standardised across participants; rather, participants wore their usual footwear. For some participants, testing was conducted using two different kinds of footwear (e.g. dress shoes and sports shoes). Footwear type was categorised by the study investigators as either: cushioned sports shoes, surgical/orthopaedic shoes, work boots, or casual/non-cushioned sneakers. In order to conduct the in-shoe testing, participants wore a waist belt which contained a wireless telemetry unit and a battery. Once fitted with the insoles and waist belt, participants were instructed to walk a distance of approximately 15 m, before turning and returning to the place where they started. Insole pressure data were collected during both of these walks, ensuring data for at least 20 steps were collected from each participant.

The time taken (in minutes) to conduct the data collection component of the study was measured from the time that the patient arrived at the pedobarography station to time of their departure, including study explanation and consent process.

### Post-pedobarography discussion with participants

At the conclusion of platform and/or insole pressure measurements, the collected trials were averaged within the relevant Novel software before being presented to the participant. The mean peak pressure (MPP) for each foot was used as the primary outcome for the purposes of data interpretation. Members of the study team with expertise in pedobarography (UK, DR) interpreted the collected trials for the participant, and answered any questions that the participant asked.

Following data interpretation, the participant was asked a small number of pre-set questions (specifically designed for this study) regarding their experience during data collection. These questions included a) whether the participant enjoyed their experience; b) whether the participant would participate again if the test was available as part of their regular clinical care; c) whether there were aspects of the test that were difficult or annoying; and d) whether the information received regarding their plantar pressures was useful (and if so, how). Information was also gathered regarding the type of footwear worn to the clinical appointment, and participants were asked what footwear they most commonly wore.

### Data management and analysis

Averaged pedobarography data were not formally analysed in terms of quantitative factors such as peak pressure and maximum force; rather, they were used to generate images for the purposes of presentation and interpretation with participants and clinicians. They were also used to provide the case study examples described later in this manuscript.

Self-report data were collected on paper by the pedobarography team, and transferred to Microsoft Excel 2010. Crude descriptive results (including proportions, %) were generated for relevant data.

Ethical approval for this study was sought and received from the University of Otago Human Ethics Committee (reference #: HE16/007), as well as local authorisation from the two District Health Boards within which the study was operating (Northland and Counties-Manukau).

## Results

### Participant characteristics

The median age of the 38 participants was 57 years (interquartile range [IQR]: 51.5–65.5 years). Participants were most commonly Māori (*n* = 15; 39% of participants), followed by New Zealand European (*n* = 9; 24%), Pacific Island (*n* = 8; 21%) and non-Māori/Pacific/European (*n* = 6; 16%).

In terms of the clinic type that participants were referred from, a total of 21 were referred from high-risk/ulcer foot clinics (55% of participants), with 10 (26%) referred from moderate-risk/non-ulcer foot clinics and 7 (18%) referred from low-risk/general diabetes clinics.

### Clinical presentation and pedobarography application

Table 1 shows the major lower-limb complications experienced by patients as reported to the study team at the time of referral, grouped according to the type of clinic that the patient was referred from. The most common complications included current ulceration (*n* = 9; 24% of all participants) and partial foot amputation (*n* = 5; 13%), although the type and location of lower-limb complications was variable across participants.

**Table 1:** Patient-level listing of existing lower-limb complications and clinical application of pedobarographic measurements, stratified by clinic type

				Pedobarography type		Application category	
Clinic type	Patient #	Lower-limb complications	Clinical application of pedobarography	Barefoot	In-Shoe	Single condition	Multi- condition
High risk/Ulcer	1	Current ulcer beneath Left 3rd and 4th metatarsal heads	Assess barefoot plantar loading under healing ulcer site	●		●	
	2	Not documented	Assess in-shoe plantar loading with orthopaedic shoe and custom insoles		●	●	
	3	Functional leg length discrepancy, has Left heel raise in shoes	Assess plantar loading, particularly around heel raise		●	●	
	4	Current ulcer beneath Right forefoot	Compare in-shoe plantar loading between work boots and sports shoes, with offloading insole in both		●		●
	5	Current ulcers on medial aspects of Left and Right hallux	Assess barefoot medial plantar loading and centre of pressure line	●		●	
	6	Peripheral neuropathy	Assess barefoot loading, particularly under 1st and 5th metatarsal heads	●		●	
	7	Not documented	Compare in-shoe plantar loading between no insole and custom orthotic insole		●		●
	8	Peripheral neuropathy, severe burns under feet	General assessment of barefoot pressures, plus compare to in-shoe pressures to show benefit of orthotic shoe and insole	●	●		●
	9	Left hallux amputation	Assessment of in-shoe loading with orthotic footwear, with and without walking frame		●		●
	10	Left 3rd toe amputation, Right 2nd toe amputation	Assessment of barefoot pressures, particularly around areas of digit amputation	●		●	
	11	Current ulcer under Left forefoot; Left 2nd-4th toe amputation	Assessment of plantar offloading within surgical shoes with custom insoles		●	●	
	12	Right foot 3rd-5th toe amputation; blister on side of Right 2nd toe	Assessment of barefoot pressures, particularly around areas of digit amputation	●		●	
	13	Current ulcer under Right 1st metatarsal head	Compare in-shoes loading between Pedor (surgical offloading shoe) and ‘Crocs’ non-closed shoes		●		●
	14	Current ulcer under Right heel	Assess in-shoe loading, particularly around Right heel wound		●	●	
	15	Right hip osteoarthritis, Right knee brace, walks with stroller	Assess in-shoe loading within Pedor orthopaedic shoes		●	●	
	16	Left 2nd toe amputation, Left 3rd toe deformity	Assess barefoot loading, particularly around area of amputation	●		●	
	17	Current ulcers on medial side of Left and Right hallux	Assess barefoot loading, particularly around Left and Right hallux	●		●	
	18	Current ulcer under medial aspect of Right hallux	Assess barefoot loading under healing ulcer site	●		●	
	19	Peripheral neuropathy	General assessment of barefoot loading	●		●	
	20	Acute Charcot foot	Assess in-shoe loading with shoes and orthotic insoles		●	●	
	21	Current ulcer under Right hallux, painful and swollen Left foot	Assess barefoot loading under healing ulcer site	●		●	
Mod risk/Non-ulcer	22	Both knees partial amputation following car accident	Assess loading patterns with and without custom insoles		●		●
	23	Severely enlarged Left and Right hallux (congenital deformity)	Assess barefoot loading, particularly hallux region	●		●	
	24	Charcot deformity	Compare barefoot and in-shoe loading, show patient benefit of wearing offloading footwear	●	●		●
	25	None	General assessment of barefoot loading	●		●	
	26	Gout	Compare old orthotic insoles with new custom orthotic insole		●		●
	27	Flat feet	General assessment of barefoot loading	●		●	
	28	Severe recurrent callous under metatarsal heads	Compare barefoot loading pre- and post-callous debridement	●			●
	29	Charcot deformity; former ulcers under Right forefoot and hallux	Assess in-shoe loading under former ulcer sites and Charcot deformity		●	●	
	30	Veruca on medial aspect of Right heel	Compare barefoot and in-shoe loading, show patient benefit of wearing offloading footwear	●	●		●
	31	Left midfoot deformity	Assess barefoot loading, particularly around Left midfoot deformity	●		●	
Low risk/General	32	Peripheral neuropathy	General assessment of barefoot loading	●		●	
	33	None	General assessment of barefoot loading	●		●	
	34	None	General assessment of barefoot loading	●		●	
	35	Arthritic pain in feet	General assessment of barefoot loading	●		●	
	36	None	General assessment of barefoot loading	●		●	
	37	Right foot pain under forefoot, callus under Right metatarsal heads	General assessment of barefoot loading	●		●	
	38	Pain under Left and Right Forefoot	Assess barefoot loading under painful Left and Right forefoot	●		●	

Table 1 also shows the primary clinical application of the pedobarographic testing for each patient, as discussed with the clinician at the time of referral. The most common applications included assessments of loading around current ulcer sites (*n* = 9; 24% of all participants), and more general/non-specific assessments of barefoot loading (*n* = 9; 24%).

Pedobarographic assessments involved either barefoot assessments with the Novel Emed system (*n* = 22; 58% of all participants; Table 1), in-shoe assessments with the Novel Pedar system (*n* = 16; 34%), or a combination of the two (*n* = 3; 8%). Based on the primary clinical applications for which the pedobarographic testing was used, we were able to categorise patients according to whether their assessment involved a single condition (*n* = 28; 74%) or multiple conditions (*n* = 10; 26%). For example, the participant for whom barefoot assessments were performed both before and after callous debridement was categorised as undergoing multiple conditions.

**Fig. 1:**
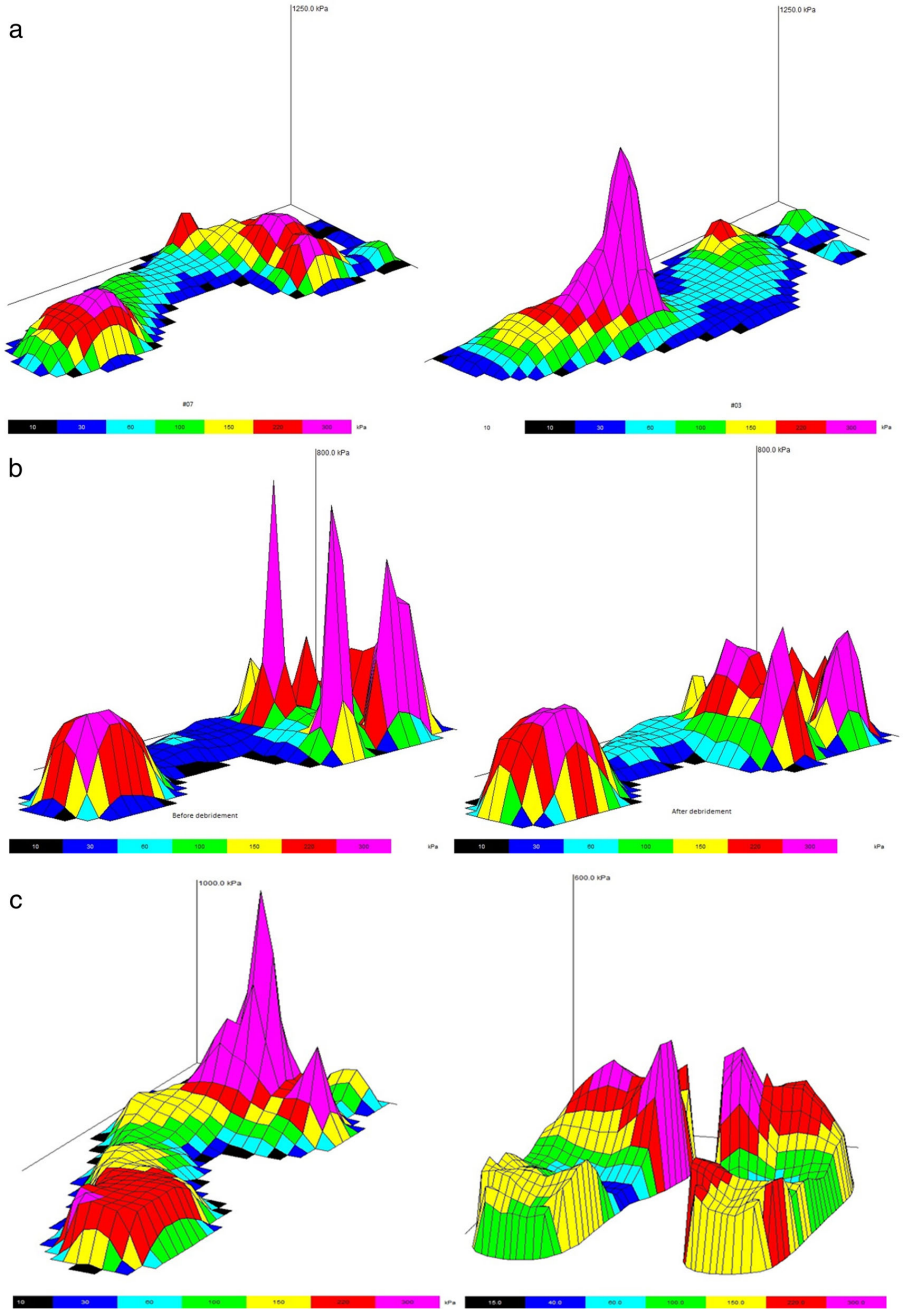
Examples of clinical applications of pedobarographic measurements; **a** barefoot pressure measurements from Patient #12, showing extreme midfoot loading under the Right foot indicative of an undiagnosed Charcot deformity; **b** barefoot pressure measurements from Patient #28, showing plantar loading beneath metatarsal heads before (*left*) and after (*right*) callous debridement; **c** barefoot and in-shoe measurements from Patient #30, showing extreme barefoot loading (left foot used as exemplar) that was significantly attenuated by the introduction of cushioned footwear and a custom insert with forefoot padding, as measured with the in-shoe system (*right*)

The median time taken to conduct the pedobarographic testing was 25 min (IQR = 20–30 min), including the time taken to explain the study and gain informed consent. The time taken to conduct the testing did not meaningfully differ depending on whether barefoot measurements (*n* = 22; median = 25 mins, IQR = 20–30 min), in-shoe measurements (*n* = 13; 25 mins, IQR = 20–34 min) or both (*n* = 3; 20 mins, IQR = 15–25 min) were conducted, nor whether a single condition (*n* = 28; 25 min, IQR = 20–30 min) or multiple conditions (*n* = 10; 21 min, IQR = 20–30 min) were conducted. Numerous observations of potential clinical importance were made during pedobarographic assessments, and we have detailed three examples in Fig. 1.

### Participant experience

Table 2 shows data relating to the participant’s experience of the pedobarography assessment. When asked after the pedobarography assessment and data interpretation if they had enjoyed their experience, all participants said yes (*n* = 38; 100%). When asked if they found any aspects of the study annoying or frustrating, most participants said no (*n* = 30; 79%). The reasons given by the *n* = 5 (13%) participants who said they found any aspect annoying or frustrating included: awkwardness/embarrassment walking in an open area, experience of back pain during walking, tiredness from walking too much, the time taken to participate and difficulty walking in a straight line. When asked if they would take part in pedobarographic assessments if they were offered to them in the future, the vast majority of participants said yes (*n* = 31; 82%). When asked if they had found the information given to them following their assessment useful, most participants said yes (*n* = 34; 89%). The reasons given for why these participants found the information useful are provided in the Additional Material (Additional file 1).

**Table 2:** Self-reported patient experience of pedobarographic testing

Measure of patient experience	Patients	
	n	%
Time taken to perform pedobarography (minutes)^a^		
Median (IQR)	25 (20–30)	
Range	15–40	
Post-Testing Questions to Participants		
Did the patient enjoy the test?		
Yes	38	100%
No	0	0%
Don’t know	0	0%
Were there any parts that were annoying or frustrating?		
Yes	5	13%
No	30	79%
Don’t know	3	8%
If this test was offered to you again, would you do it?		
Yes	31	82%
No	0	0%
Don’t know	7	18%
Did you find the information useful?^b^		
Yes	34	89%
No	0	0%
Don’t know	3	8%

^a^Time from patient arriving at pedobarography station to time of their departure, including study explanation and consent process

^b^Question asked following explanation and interpretation of pedobarography observations with biomechanics experts (DR, UK)

### Footwear behaviour

The footwear worn by participants to their clinical appointment is shown in Table 3. Footwear varied considerably and was patterned by clinic type. Across the total group, the majority of participants wore closed footwear to their appointment (*n* = 23; 71% of all participants). The most common closed footwear type worn by participants was surgical/orthopaedic footwear (*n* = 11; 29%).

**Table 3:** Footwear-related behaviour for total sample and by clinic type

			Patients, by clinic type					
Footwear behaviour	Total patients		High risk/ulcer		Mod. risk/non-ulcer		Low risk/general	
	n	%	n	%	n	%	n	%
Patient wearing closed footwear to clinic	27	71%	16	76%	7	70%	4	57%
Cushioned sports shoes	9	24%	4	19%	4	40%	1	14%
Surgical/Orthopaedic shoes^a^	11	29%	10	48%	1	10%	0	0%
Work boots	3	8%	1	5%	1	10%	1	14%
Casual/non-cushioned sneakers	4	11%	1	5%	1	10%	2	29%
Patient wearing non-closed footwear to clinic^b^	11	29%	5	24%	3	30%	3	43%

^a^Surgical/orthopaedic shoes were primarily Pedor Stretch diabetic orthopaedic shoes

^b^non-Closed footwear included flip-flops (*n* = 5), sandals, (*n* = 1), slides (*n* = 2), ‘Crocs’-style shoes (*n* = 2) and Mary Janes (*n* = 1)

When examining footwear behaviour by clinic type, those attending high-risk foot clinics were most likely to wear closed footwear (*n* = 16; 76% of high-risk participants), followed by those attending moderate-risk clinics (*n* = 7; 70% of moderate-risk participants) and then those attending low-risk clinics (*n* = 4; 57% of low-risk participants). The most common closed footwear worn by those attending high-risk foot clinics were surgical/orthopaedic footwear (*n* = 10; 48%).

## Discussion

The aim of this study was to assess the feasibility of incorporating pedobarographic testing into the clinical care of diabetic feet in New Zealand. Specifically, we aimed to assess a) the response rate among patients invited to take part in pedobarographic testing; b) the extent to which additional testing interrupts normal clinic time; c) the experience of patients who took part in pedobarographic testing; d) the applications of the pedobarographic testing that were most useful to those clinicians in charge of diabetic foot care; and finally e) which aspects of the pedobarographic intervention could feasibly be tested in a clinical trial.

### Response rate

In terms of response rate, we observed that the vast majority of those patients invited to participate agreed to do so (81%). The high response rate is perhaps unsurprising, for two reasons: firstly, the study was introduced to the patient by their clinician – most of whom were co-investigators on the study. Secondly, participation in the study did not require participants to travel elsewhere to take part or return for later assessment, since participation took place directly following the patient’s usual clinical appointment.

### Disruption to clinics

With regards to disruption to normal clinic time, all but one of the participants underwent pedobarographic testing after their normal clinical appointment, and thus the normal clinical appointment was not interrupted. We observed that the pedobarography test was relatively quick to perform. Even when including those participants for whom multiple conditions were tested, the median duration of the test was 25 min – including the time taken to explain the test to participants and gain informed consent. The participants were overwhelmingly positive about their experience during the study – with all stating that they enjoyed their experience, and most (89%) stating that they found the information they received at the conclusion of the test useful.

### Patient complexity

In general, most of the patients referred into the study were clinically complex. Most suffered multiple lower-limb complications (some unrelated to their diabetes), and many had already undergone partial foot amputation. While all but one patient was able to complete the walking required to collect the pedobarographic data, several patients had limited mobility – in which case the in-shoe system was preferred, since less walking is required with this system in order to gain sufficient data. Only two of the patients who participated in the study usually walked with either a cane or a walking frame, although both were able to comfortably walk without this support. The use of a cane or walking frame has ramifications in terms of plantar loading, in that the offloading achieved via the use of these devices is likely to reduce loading under the feet. While it is important to be aware of this likelihood, measurements taken from this population are still meaningful – in that they still represent the usual plantar loads experienced by that patient while walking (assuming the device is used during the majority of the patient’s ambulation). It is also important to note that relative comparisons between conditions (e.g. custom orthoses compared to standard insole) would also be unaffected by the use of mobility support.

### Adverse events

Despite working with a high-risk population, there were no adverse events in this study – an observation which is in-keeping with the low-risk nature of the medical device(s) used during the study. The greatest negative impact on patients was likely the time taken to participate (although only one of the five patients who stated that they found some aspect of the study annoying or frustrating identified the time taken to collect data as their key annoyance/frustration). As a means of combatting this, it would have been useful to conduct pedobarographic testing during a separate appointment, rather than as an optional addition to an existing appointment. This would have had the added benefit of warning participants in advance about the need to bring items such as their normal footwear and offloading devices, and also assist the research team in preparing for a patient’s arrival – which, on occasion, was difficult when patients were being referred from several clinics simultaneously. However, it was not pragmatically possible to book appointments for patients ahead of time for the current feasibility study.

### Clinical application of pedobarography during the study

The most common reason that patients were referred into the study by clinicians was to assess plantar loading around a site of a current or previous ulcer, with nearly a quarter (24%) of all participants referred for this reason. In most instances, we were able to observe the degree to which offloading had been achieved with custom orthoses (Table 1). This information was fed-back to the patients and, where possible, also to the referring clinician. Our ability to compare in-shoe offloading interventions – interventions generally provided with little idea of the degree to which offloading is actually being achieved – was the most popularly-applied example of a clear and feasible means by which pedobarography could be integrated into diabetic foot care in New Zealand.

The value of general foot screening among those patients referred from general diabetes clinics was less clear. Seven of those patients who participated in the current study (18% of participants) were referred by either diabetes nurse specialists or dieticians, and most of these patients had minor (if any) lower-limb complications. Because of this, participation was unlikely to result in meaningful information that might affect the foot care of these patients in the short- to medium-term. However, all (100%) of these participants still reported that they found the information useful; and when asked what they found most useful, several stated that the test had made them ‘more aware’ of their feet (Additional file 1). We may cautiously extrapolate from this observation that the test may have positively impacted the foot-related health of the patient in the medium- to long-term, by providing them with some education about the importance of plantar pressure, the choice of appropriate footwear and taking care of their feet; however, this is purely speculative. Given the absence of evidence that early intervention with pedobarography improves diabetic foot outcomes among low-risk patients, further work is required to understand the possible benefits (and harms) of pedobarographic measurements in this population.

### Application of pedobarography in other international contexts

To date, the application of pedobarography to diabetic foot care has fallen into two categories: 1) as a means of predicting the risk of ulceration [35, 47]; and 2) as a means of informing the construction of offloading orthoses [32–34, 48, 49]. With respect to the former, Pham et al. [35] compared the value of multiple clinical markers (including peripheral sensation, vibration perception, a neuropathy disability score, and barefoot peak plantar pressure) for predicting whether a patient would sustain a foot ulcer within a period of several years. The authors observed only moderate sensitivity (59%) and specificity (69%) when the test was used only by itself; however, specificity improved somewhat (up to 78%) when the plantar pressure data were combined with the neuropathy disability score. Similarly, Lavery et al. [47] investigated the usefulness of barefoot pressure measurements as a means of predicting which of the patients that presented to a diabetes outpatient clinic would ulcerate. These authors also observed only modest sensitivity and specificity when pedobarography was used on its own as a predictive tool [47]. However, as noted by Bus [50], the predictive value of in-shoe pedobarographic measurements remains unexplored. A key challenge to the viability of using pedobarography as an ulcer-prediction tool is the absence of a widely-used, validated threshold of peak plantar pressure beyond which a patient is likely to be at increased risk of ulceration.

While the predictive value of pedobarography in terms of plantar ulceration remains uncertain, the value of this tool in guiding clinicians to the effective management of current (or previous) ulcers is more convincing. Owings et al. [34] observed that insoles created with the support of pedobarographic information resulted in a 32% and 21% reduction in peak pressure compared to two insoles (respectively) that were independently constructed without this information [34]. Ulbrecht et al. [33] observed that patients for whom insole design was not guided by barefoot pressure measurements were nearly three-and-a-half-times more likely to sustain an ulcer compared to a group of patients for whom this information was collected (adjusted hazard ratio [HR]: 3.4, 95% CI 1.3–8.7) [33]. Bus et al. [49] used in-shoe pedobarographic measurements to optimise footwear modifications among neuropathic diabetic patients, and successfully reduced plantar pressure by nearly a third (30%) across the cohort using this information. In a separate study, Bus et al. [32] observed that among patients with a recently-healed plantar ulcer who had high adherence to their treatment, the use of in-shoe pressure measurements to guide the modification of custom footwear substantially reduced the risk of ulcer reoccurrence compared to those who received custom footwear that was not modified based on this data (re-ulceration rate: 26% pedobarography group, 48% usual care group; odds ratio 0.38, 95% CI 0.15–0.99) [32].

When speaking about the role of pedobarography in guiding the care of high-risk diabetic feet, Bus [50] recently wrote: “*This is a major innovation for footwear prescription practice, which has traditionally been more of an art than a science, where footwear was designed and evaluated based on the expertise, skills and experience of the prescribing physician and shoe technician, and efficacy was judged by whether a foot ulcer developed or not.*” Based on the recent evidence detailed above, and our own observations made during this feasibility study, the efficacy of pedobarography in the reduction of diabetic foot morbidity in New Zealand deserves further examination via clinical trial.

### Observations regarding clinical trial development

In terms of a clinical trial development, the current study leads us to believe that there are two (non-mutually exclusive) avenues that deserve further investigation: a) the use of pedobarography to inform the design and effectiveness of offloading devices among high-risk diabetic patients, as has been performed elsewhere (but not with a focus on the specific needs in the New Zealand population); and b) the use of pedobarography as a means to increase offloading footwear and/or orthosis compliance among high-risk diabetic patients (including patients with current ulceration). Both of these investigations may only require an in-shoe pedobarography system, since this system can be readily used to measure the effectiveness of interventions in high-risk populations. There may be some benefit in using barefoot measurements to assist with the creation of custom insoles, as has been performed in the past [33, 34]; however, it is also possible that the in-shoe system may provide the necessary information to assist with reductions in plantar loading [32, 49].

The current feasibility study taught us numerous lessons regarding the logistical operation of a full clinical trial. For example, nearly all patients in the study underwent pedobarographic testing at the conclusion of their clinical appointment. As such, it was difficult to immediately inform foot care when the patient was not going to return to the referring clinician after the pedobarography test (commonly, the referring clinician had already moved on to their next patient). A trial that formally aimed to evaluate the usefulness of pedobarography in informing diabetic foot care in New Zealand should consider a) which pedobarographic information needs to be collected from the patient (including condition comparison, such as custom insoles vs. standard insoles, or other pressure-reducing interventions such as debridement and hosiery); b) who needs this information (e.g. an orthotist, if informing the design of offloading orthoses); and c) when this person needs this information (e.g. immediately so that an offloading intervention can be customised before the patient leaves the clinic). Again, collecting this information during a separate appointment (which could be immediately after the normal clinical appointment) is appealing, since it would allow for flexibility in terms of condition comparison and immediate feedback to relevant parties.

### Footwear

The majority (71%) of patients wore at least some form of closed footwear to their appointment, while 29% wore non-closed footwear (such as flip-flops); however the type of footwear worn to a clinical appointment is not necessarily indicative of chronic footwear behaviour. For example, it is feasible that patients are more likely to wear the footwear that they believe will be viewed most favourably by their podiatrist (in the case of those attending foot clinics).

Data regarding footwear behaviour in New Zealand – and, of particular relevance, how this behaviour is patterned by demographic characteristics including ethnicity – is scarce; however, some authors have suggested that the wearing of non-closed footwear is common in New Zealand [51]. Given a) the importance of offloading footwear and insert interventions, and b) the possibility that cultural commonalities may reduce adherence to closed footwear (particularly during the warm summer months [52]), further prospective research is required around footwear compliance in this population – since the efficacy of any offloading intervention among high-risk diabetic patients is entirely contingent on habitual adherence [32].

### Limitations

The accuracy of the response rate achieved in this study relies on self-report from the referring clinicians, who were asked to report to the research team when patients declined to participate. It is feasible that, amidst a busy clinical environment, some clinicians did not report some declines – in which case, the high response rate gained in the current feasibility study may be an exaggeration. On the other hand, given that the main reason patients gave for declining was time restriction (e.g. needing to leave immediately following the appointment to return to their job), it is possible that the response rate would have been higher than observed had patients been warned about the study in advance. On balance, we believe that the response rate achieved is a relatively accurate reflection of the popularity of the pedobarographic test, and reflects the likely response rate that might be achieved in a full clinical trial.

The questions asked of participants regarding their experience during data collection were not based on any previously validated questionnaires, and largely involved dichotomous (yes/no) responses (rather than Likert scales). This was because we were unable to find a validated questionnaire that addressed the relevant topics.

## Conclusions

The current feasibility study embedded pedobarographic measurements into multiple unique diabetic foot clinic settings in the New Zealand context, and observed a high response rate and positive self-reported experience from participants. With regards to disruption to normal clinic time, the median time for pedobarographic testing (including study introduction and consenting) was 25 min. All but one of the participants underwent pedobarographic testing after their normal clinical appointment. As part of our engagement with participants, we observed a high degree of lower-limb morbidity, including current ulceration and chronic foot deformities. Despite working with a high-risk population, there were no adverse events in this study – an observation which is in-keeping with the low-risk nature of the medical device(s) used during the study. In terms of application of pedobarography as a clinical tool in the New Zealand context, the current feasibility study leads us to believe that there are two avenues that deserve further investigation: a) the use of pedobarography to inform the design and effectiveness of offloading devices among high-risk diabetic patients; and b) the use of pedobarography as a means to increase offloading footwear and/or orthoses compliance among high-risk diabetic patients. Both of these objectives deserve further examination in New Zealand via clinical trial.

## Additional file

Additional file 1: Free-text responses from patients to the following post-testing question: “What part of the information [from the pedobarography results] did you find useful?”. (DOCX 17 kb)
