# Supplementary material for: Pedobarography as a clinical tool in the management of diabetic feet in New Zealand: a feasibility study
Source: J Foot Ankle Res. 2017 Jun 9;10:24. doi: 10.1186/s13047-017-0205-6 (PMC5466715; doi:10.1186/s13047-017-0205-6)
Supplement: Additional file 1: — Free-text responses from patients to the following post-testing question: “What part of the information [from the pedobarography results] did you find useful?”. (DOCX 17 kb) [file 13047_2017_205_MOESM1_ESM.docx]

# Additional file

**Additional file 1:** *Free-text responses from patients to the following post-testing question: “What part of the information [from the pedobarography results] did you find useful?”*

*High risk group:*

- Patient #1: “[The information] will help me choose footwear. I am keen to get orthotics that reduces the pressures [under the healing ulcer site].”
- Patient #3: “[It was interesting] to see the differences between [my] legs and feet…to see what’s happening. I will try to use the other leg more.”
- Patient #4: “It was useful to see that the cut made by [the podiatrist] in the insole…showed that pressures decreased [in that area].”
- Patient #5: “[The information] makes me want to keep my feet safe.”
- Patient #6: “[It was interesting] to find out more about what is happening [to my feet].”
- Patient #7: “[It was interesting] to see difference between inserts and no [inserts]….to see what is happening to my feet.”
- Patient #8: “[It was interesting] to see what is happening with the pressures.”
- Patient #9: “[That is] a hard question.”
- Patient #10: “[The information is useful] as long as it helps others.”
- Patient #11: “Seeing the stress distribution was useful.”
- Patient #13: “[The information] is more for the clinicians, as they may make something of it.”
- Patient #14: “[The information] made me mindful of what is going on in terms of how I am loading my feet.”
- Patient #15: “[The information] is useful as long as it helps others.”
- Patient #16: “I knew that I had different loading between my feet…[so that was] good to see. I was unaware of high loading under [my] forefoot and toes.”
- Patient #18: “It was good to see what was happening.”
- Patient #19: “[It was interesting] to see usefulness of insoles…visuals are important….lots of people don’t respond to hearing things.”
- Patient #21: “[The software has] a simple design, good visuals. [It was] good to see the difference between feet.”

*Moderate risk group:*

Patient #22: “[The information is only] useful if it helps with my care.”

Patient #24: “[The information] was interesting.”

Patient #26: “[It was] interesting to see loading under [my] feet…[I see] a lot of value in having this as a monitoring tool.”

Patient #30: “[It was] good to see areas of high pressure and to talk about how to [improve] them.”

Patient #31: “[It was] very interesting, very quick…so good to see what is happening…I suspected it would be this way.”

*Low risk group:*

Patient #32: “[Patient speaking through interpreter:] I feel like I am being taken care of.”

Patient #33: “Good to know what is going on [with my feet].”

Patient #34: “[It was useful] because the pressures were good…if the pressures were bad, then I would have been worried.”

Patient #35: “[It was] interesting to compare feet [and to] see the flatter foot.”

Patient #37: “[It was interesting] to see pressures under areas where I am having pain.”

Patient #38: “[It was interesting] to see the pressures under areas of [my] feet where I am experiencing pain.”
